# Manual versus automatic indices assessment based on a marker-less, three dimensional, structural light surface topography postural evaluation. Preliminary study

**DOI:** 10.1186/1748-7161-8-S1-O24

**Published:** 2013-06-03

**Authors:** W Glinkowski, J Michoński, B Glinkowska, R Sitnik

**Affiliations:** 1 Department of Orthopaedics and Traumatology of Locomotor System, Center of Excellence "TeleOrto", Medical University, Warsaw, Poland; 2 Institute of Micromechanics and Photonics, Warsaw University of Technology, Warsaw, Poland; 3 Department of Sport and Physical Education, Medical University of Warsaw, Warsaw, Poland

## Background

Posterior Trunk Symmetry (POTSI) and Deformity in the Axial Plane (DAPI) Indices, and sagittal plane curvature measurements, are frequently used to measure deformities.

## Aim

The aim of this study was to perform a preliminary assessment, and a reliability test, of the algorithm designed to provide a more reliable way of localizing anatomical back shape landmarks for school screening subjects.

## Methods

A 3D Orthoscreen system, designed for postural deformity telediagnostics, using the structural light method, was used for school screening [2,3]. Clouds of dots were acquired for every subject. POTSI, DAPI, kyphosis and lordosis were measured, after marker-less landmarking of anatomical back structures, manually and automatically, for the same subjects. 50 subjects were assessed, 22 male adolescent subjects (average age 15.18 years - from 14 to 17) and 28 females (average age 14.55 - from 14 to 16) were examined. Their clouds of dots were acquired and saved. Clouds were retrieved and analyzed utilizing 3D Orthoscreen system. An operator (trained professional) retrieved 3D data and analyzed by pointing landmarks manually or automatically. After landmarking, procedure Indices and angular measurements were calculated by the system. MedCalc statistical software (Version 12.1.4.0) was used to calculate Inter-rater correlation for measurements.

## Results

Intra-class correlation coefficients, for manual and automatic raters, were calculated for all assessed parameters. Strong, and almost perfect, agreement was found for sagittal plane curvature measurements (kyphosis and lordosis). The Intra-class correlation at 95% Confidence Interval for Kyphosis, Automatic vs. Manual measurements, for average measures was 0.87 (from 0.77 to 0.92), and Lordosis, Automatic vs. Manual measurements, for average measures 0.958 (from 0.9261 to 0. 9761), POTSI, Automatic vs. Manual, for average measures 0.72 (from 0.49 to 0.84). ICC for DAPI Automatic vs. Manual measurements was 0.02 – 0.047.

## Conclusion

Strong, and almost perfect, agreement we found for sagittal plane curvature measurements (kyphosis and lordosis) may coincide with the method of measurement (Debrunner like method). Fair agreement for POTSI Index, and poor for DAPI, was found in this preliminary series. It may depend on the formula used for Index's calculation. Further studies, and development of the algorithm, and source of inadequacies, shall be considered to improve automatic calculation reliability.
